# Biallelic Deletion of *Pxdn* in Mice Leads to Anophthalmia and Severe Eye Malformation

**DOI:** 10.3390/ijms20246144

**Published:** 2019-12-05

**Authors:** Hyun-Kyung Kim, Kyung A Ham, Seung-Woo Lee, Hong Seok Choi, Hong-Sug Kim, Hong Kyung Kim, Hae-Sol Shin, Kyoung Yul Seo, Yejin Cho, Ki Taek Nam, In-Beom Kim, Young Ae Joe

**Affiliations:** 1 Cancer Research Institute, College of Medicine, The Catholic University of Korea, Seoul 06591, Korea; genesis1109@catholic.ac.kr (H.-K.K.); hga9409@naver.com (K.A.H.); mrkid2@naver.com (S.-W.L.); sody132@naver.com (H.S.C.); 2 Department of Medical Life Sciences, College of Medicine, The Catholic University of Korea, Seoul 06591, Korea; 3 Department of Biomedicine & Health Sciences, The Catholic University of Korea, Seoul 06591, Korea; 4 Department of Genetic Engineering Mouse, Macrogen Inc, Seoul 08511, Korea; khongsug@macrogen.com; 5 Korea Mouse Sensory Phenotyping Center (KMSPC), Yonsei University College of Medicine, Seoul 03722, Korea; THBIOMAN@yuhs.ac (H.K.K.); HSOL916@yuhs.ac (H.-S.S.); SEOKY@yuhs.ac (K.Y.S.); 6 Institute for Vision Research, Department of Ophthalmology, Yonsei University College of Medicine, Seoul 03722, Korea; 7 Severance Biomedical Science Institute, Brain Korea 21 PLUS Project for Medical Science, Yonsei University College of Medicine, Seoul 03722, Korea; MAMAYJ87@yuhs.ac (Y.C.); KITAEK@yuhs.ac (K.T.N.); 8 Department of Anatomy, College of Medicine, The Catholic University of Korea, Seoul 06591, Korea; ibkimmd@catholic.ac.kr

**Keywords:** peroxidasin, knockout mice, anophthalmia, microphthalmia, eye development, CRISPR/Cas9

## Abstract

Peroxidasin (PXDN) is a unique peroxidase containing extracellular matrix motifs and stabilizes collagen IV networks by forming sulfilimine crosslinks. PXDN gene knockout in *Caenorhabditis elegans* (*C. elegans*) and *Drosophila* results in the demise at the embryonic and larval stages. PXDN mutations lead to severe eye disorders, including microphthalmia, cataract, glaucoma, and anterior segment dysgenesis in humans and mice. To investigate how PXDN loss of function affects organ development, we generated *Pxdn* knockout mice by deletion of exon 1 and its 5′ upstream sequences of the *Pxdn* gene using the CRISPR/Cas9 system. Loss of both PXDN expression and collagen IV sulfilimine cross-links was detected only in the homozygous mice, which showed completely or almost closed eyelids with small eyes, having no apparent external morphological defects in other organs. In histological analysis of eye tissues, the homozygous mice had extreme defects in eye development, including no eyeballs or drastically disorganized eye structures, whereas the heterozygous mice showed normal eye structure. Visual function tests also revealed no obvious functional abnormalities in the eyes between heterozygous mice and wild-type mice. Thus, these results suggest that PXDN activity is essential in eye development, and also indicate that a single allele of *Pxdn* gene is sufficient for eye-structure formation and normal visual function.

## 1. Introduction 

Basement membranes (BM) are sheet-like, cell-adherent extracellular matrices (ECM) consisting of an enmeshed polymer of laminins and collagen IV (Col IV) that are bound to nidogens, agrin, and perlecan [1]. They serve as both supportive cell substrata and solid-phase agonists, contributing tissue organization, stability, and differentiation. Col IV is essential for basement membrane (BM) stability [2], and laminins are critical to the general scaffolding of BM. Col IV is assembled by extracellular oligomerization of triple-helical protomers and is covalently cross-linked by sulfilimine (S=N) bond formation among the C-terminal non-collageneous 1 (NC1) domains that reinforce the structural integrity of the Col IV networks [3]. Ablation of both Col IV subunit α1 and α2 in mice causes lethality between E10.5 and E11.5 because of structural deficiencies in the BMs and eventual failure of the integrity of Reichert’s membrane [2]. A dominant mutation of Col IV subunit chain α1 in mice results in BM defects that affect the eye, kidney, and other tissues [4]. In humans, Col IV α1 mutation is also associated with abnormalities of the anterior chamber of the eye, vascular leukoencephalopathy, and hemorrhagic stroke [5,6,7]. Col IV α2 mutations in mice and humans also cause diseases similar to those resulting from Col IV α1 mutations [8,9].

Peroxidasin (PXDN) is an ECM protein with peroxidase activity, which catalyzes formation of the sulfilimine bond between a methionine sulfur and hydroxylysine nitrogen using hypohalous acids in Col IV [10]. Loss or decrease of PXDN severely disrupts organogenesis in *Drosophila*, *C. elegans*, and zebrafish by impairing basement integrity, leading to death at embryonic and larval stages (*Drosophila and C. elegans*) or severely defective phenotypes, which consist of cardiac edema, decreased eye size, and gross trunk patterning defects (zebrafish) [10,11,12]. The sulfilimine bond of Col IV is considered to contribute to reinforcing the mechanical strength of BM and maintaining tissue integrity. In mice and humans, much as in Col IV mutations, it can be postulated that PXDN mutations can cause BM-linked diseases, more specifically, Col IV-associated diseases, such as porencephaly-1, intracerebral hemorrhage, retinal arterial tortuosity, or anterior segment dysgenesis (ASD) [4,13]. Indeed, some homozygous mutations in *PXDN* in humans cause severe inherited eye disorders, such as congenital cataracts, corneal opacity, and developmental glaucoma because of ASD; other recessive mutations in *PXDN* show a broader phenotype, including ASD, sclerocornea, microphthalmia, hypotonia, and developmental delays [14,15]. *Pxdn* mutation-induced congenital eye diseases, such as ASD and microphthalmia, were also revealed in recessive mutant mice induced by *N*-ethyl-*N*-nitrosourea (ENU) [16]. The mutant mice highly resemble the manifestations in patients with *PXDN* mutations. However, these pathogenic phenotypes are milder than expected from the studies of lower animals, in which PXDN depletion causes embryonic and larval lethality or severely defective phenotypes. In addition, it has not been established yet how inactivation of the PXDN gene affects tissue genesis and organ development. 

Here, we generated a knockout mouse model by deletion a sequence containing exon1 and its 5′ upstream sequences of the *Pxdn* gene using a CRISPR/Cas9-based genome editing system and analyzed the phenotypes of homozygous/heterozygous mice. We found that the homozygous mice had no eyes or extremely disorganized eye structures, whereas the heterozygous mice underwent development of normal eyes with proper visual functions. Grossly, no external morphological defect was detected in other organs. This study provides experimental evidence that PXDN is critical for eye development, especially in constructing precise eye structures, and that PXDN is haplosufficient for eye structure formation and normal visual function. This study also suggests that *Pxdn* knockout mice can be used as a novel mouse model for anophthalmia and severely malformed microphthalmia. 

## 2. Results

### 2.1. Generation of Pxdn Gene Knockout Mice

**Figure 1:**
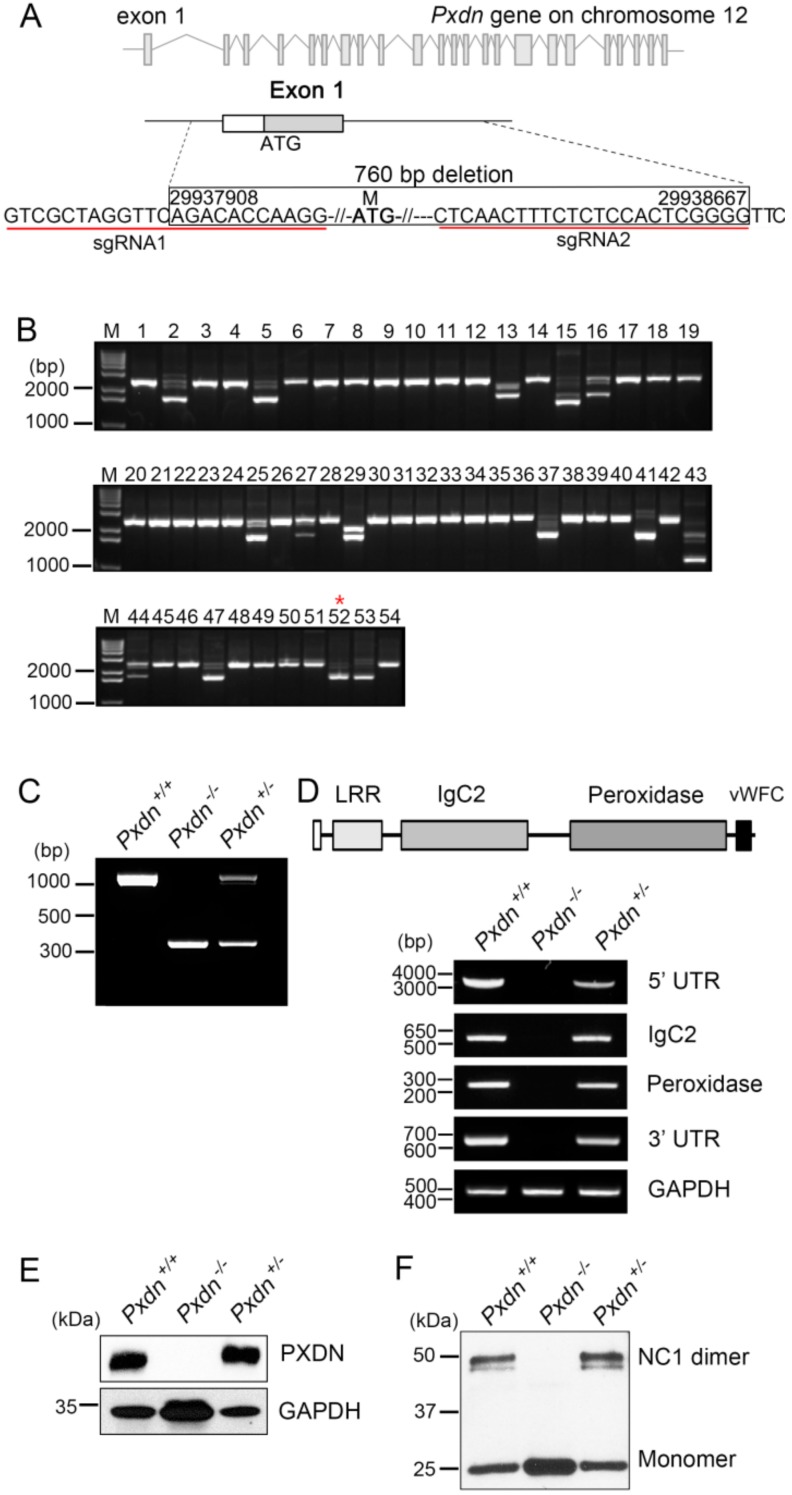
Construction of mice with deletion of the exon1 and its 5′ upstream sequences of the peroxidasin *(Pxdn)* gene. (**A**) Genomic locus of mouse *Pxdn* gene on chromosome 12 and cutting sites of sgRNAs. A total of 760 bps encompassing exon1 and its 5′ upstream sequences of the *Pxdn* gene were deleted by the CRISPR/Cas9 system. (**B**) Genotyping of F0 mice (#1–#54). PCR was performed using genomic DNA isolated from tail-cut samples and a primer pair, SF3 and SR3. The #52 mouse (asterisk) among deletion mutants had 760 bps deletion containing exon1 and its 5′ upstream sequences of the *Pxdn* gene. (**C**) The genomic DNA isolated from the lung tissues of the progeny mice was used for PCR analysis using primer pairs SF1 and SR2. (**D**) A schematic depiction of PXDN with leucin-repeat-rich (LRR), immunoglobulin (Ig), peroxidase, and von-Willebrand factor type C (vWFC) domains (upper panel). Inactivation of the *Pxdn* gene was verified by RT-PCR analyses (lower panel). Total RNAs were isolated from the lung tissue of the mice and used for RT-PCR analysis using specific primers that bind to different regions of the *Pxdn* gene: 5′ UTR-exon 19, exons 10–14 (IgC2 3–4 domain), exons 17–19 (peroxidase domain), and 3′ UTR region. GAPDH was used as a loading control. (**E**) Immunoblot analysis of PXDN expression was done using the lung tissue of the mice and anti-mouse PXDN polyclonal antibody. (**F**) Immunoblot analysis of non-collageneous 1 (NC1) crosslinked dimer/un-crosslinked monomer levels of collagen IV (Col IV) using the lung tissue of the mice. The tissue lysate was treated with collagenase prior to immunoblot analysis.

To identify the function of PXDN, we attempted to generate *Pxdn* gene knockout mice by means of a CRISPR/Cas9 system using guide RNAs targeting the exon1 and its 5′ upstream sequences of *Pxdn* (locus number, NC_000078.6) (Figure 1A). We performed genotyping analysis of founder mice using tail-cut samples by PCR and Sanger sequencing. We found that some mice had variously altered sequences compared with the normal *Pxdn* sequence. Among them, we selected founders that had deletion of the exon1 and its 5′ upstream sequences of the *Pxdn* gene (760 bps) (Figure 1B, red asterisk). The *Pxdn*-deleted mutants were chosen on the basis of the analysis of hypothetical open reading frames of the prospective transcript. The 760 bps-deleted mouse (female) was bred with C57BL/6N normal strains and maintained as heterozygotes and homozygotes.

After a generation of progeny, the mutant mice were verified by PCR using genomic DNA isolated from the lung tissues. When both primers located out of the deletion region were used, as expected, wild-type (*Pxdn^+/+^*, WT) and (*Pxdn^−/−^*, KO) mice showed a single amplified DNA fragment (1096 bp for WT, 336 bp for KO), whereas two DNA fragments were amplified because of two different alleles of the *Pxdn* gene in heterozygous mice (*Pxdn^+/−^*) (Figure 1C). Next, we examined the inactivation of the *Pxdn* gene at mRNA level in the KO mice. When PCR analysis was done using various primer sets located in the region from 5′ UTR to 3′ UTR of PXDN mRNA, only WT and heterozygous mice showed the expected PCR product amplified from the PXDN mRNA, and the KO mice showed no amplified PCR product in any primer set (Figure 1D). These data show that deletion of the exon1 and its 5′ upstream sequences of the *Pxdn* gene leads to complete inactivation of the *Pxdn* gene. To evaluate *Pxdn* gene expression at the protein level, we generated a polyclonal antibody against a mouse PXDN subdomain, which was an immunoglobulin (Ig)C2 (3–4) motif within the extracellular matrix motifs of mouse PXDN. As shown in Figure 1E, PXDN was expressed in the lung tissues derived from WT and heterozygous mice. Consistent with the RNA expression pattern, the PXDN protein was not detected in the KO mice. Finally, we tested whether a known biochemical function of PXDN was lost upon exon1 and its 5′ upstream sequences deletion of the *Pxdn* gene. PXDN is a matrix peroxidase enzyme that crosslinks NC1 domains of Col IV by forming sulfilimine bonds [10]. Thus, we examined whether PXDN deficiency leads to lack of sulfilimine bond formation in the KO mice. After the lung-tissue lysate was digested by collagenase, the samples were immunoblotted using anti-Col IV α2 antibody. Expectedly, the KO mouse samples showed no NC1 dimers, but instead increased NC1 monomers, in contrast to WT or heterozygous mouse samples (Figure 1F). There was no change in dimer versus monomer levels between the WT and heterozygous mouse samples, which was consistent with the PXDN protein levels of the WT and heterozygous mice. Therefore, we concluded that the mutant mice generated from deletion of exon1 and its 5′ upstream sequences of the *Pxdn* gene show complete inactivation of the *Pxdn* gene, and that loss of PXDN leads to no sulfilimine crosslinking of the NC1 domains of Col IV.

### 2.2. External Morphological Defect Was Obvious in Eyes, but Not Other Organs in Pxdn-Null Mice 

**Figure 2:**
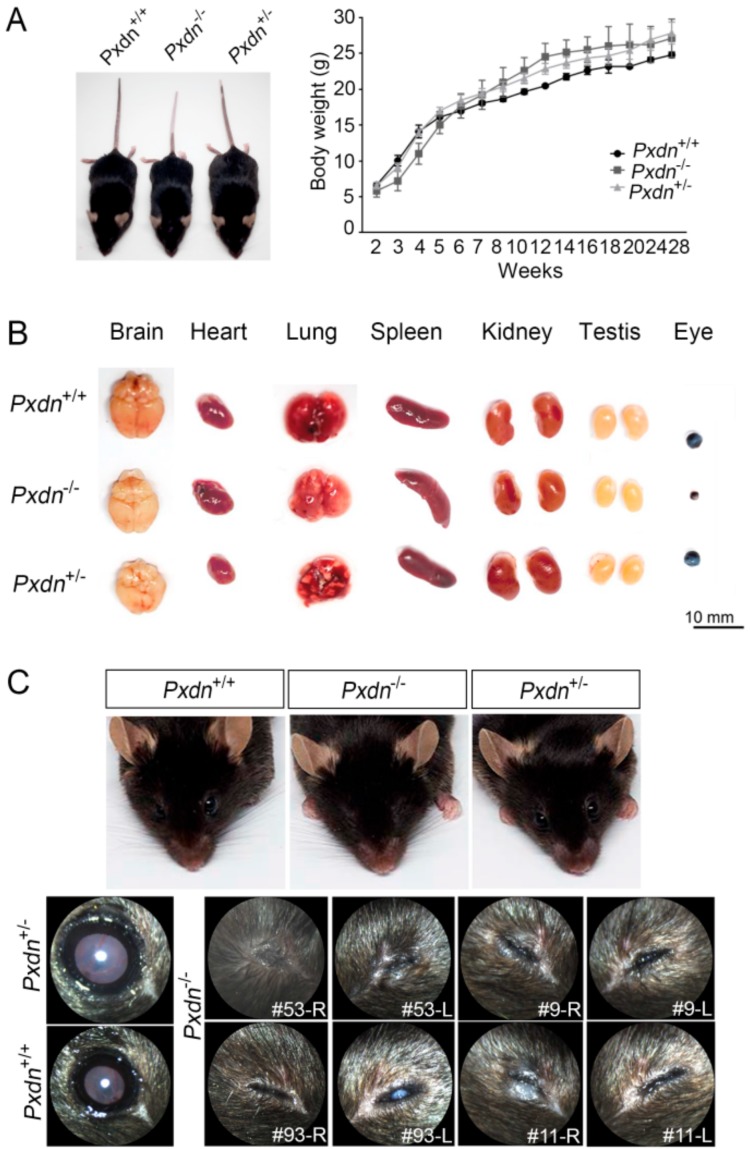
Deletion of the exon1 and its 5′ upstream sequences of the *Pxdn* gene caused congenital malformation of eyes in mice. (**A**) Representative photograph of *Pxdn*^(+/+)^ (WT), *Pxdn*^(−/−)^ (KO), and *Pxdn*^(+/−)^ (heterozygous) mice at 9 weeks of age (left). Body weight of the WT (*n* = 3), KO (*n* = 7), or heterozygous (*n* = 10) mice. (**B**) Comparison of the organs removed from the mice, which were approximately 20 weeks old. (**C**) Representative external morphology of the eyes in the WT, heterozygous, and KO mice. Compared with the WT (*n* = 34) or heterozygous mice (*n* = 115), completely or almost closed eyelids were noted in the KO mice (*n* = 40).

PXDN is expressed in endothelial cells, epithelial cells, and fibroblasts. Relatively high expression of PXDN is observed in several human tissues such as the heart, spleen, kidney, and lung [17,18]. As PXDN stabilizes Col IV, a major component of BM, its deficiency in lower animals leads to lethality because of disorganized tissue structures. However, when the *Pxdn* gene was inactivated in mice, adult mice did not show any significant change in size and weight between the WT and mutant mice (Figure 2A). In addition, these mice showed no changes in size and external morphology in most organs, including the brain, heart, lung, spleen, kidney, and testis, except for the eyes (Figure 2B). Small eyes were observed in the KO mice. In addition, as a minor change, change of hair color was noticed and the tail color was white in two-thirds of the tip of the KO mice, which also had a white spot at the ventral and dorsal region at a frequency of about 94.1%, as observed in the *Pxdn* mutant mice (*KTA048*) with a premature stop codon [16]. The KO mice showed severe abnormalities in the external appearance of the eyes compared to the WT and heterozygous mice (Figure 2C). They had completely or almost closed eyelids, and some of them (#9-L, #93-L) had severe cataracts in the eyes, whereas the heterozygous and WT mice born from the crossing had normal eyelids. Thus, we concluded that loss of PXDN leads to severe external morphological defects of the eyes.

### 2.3. Extremely Disorganized Eye Structure Was Shown in Pxdn-Null Mice

**Figure 3:**
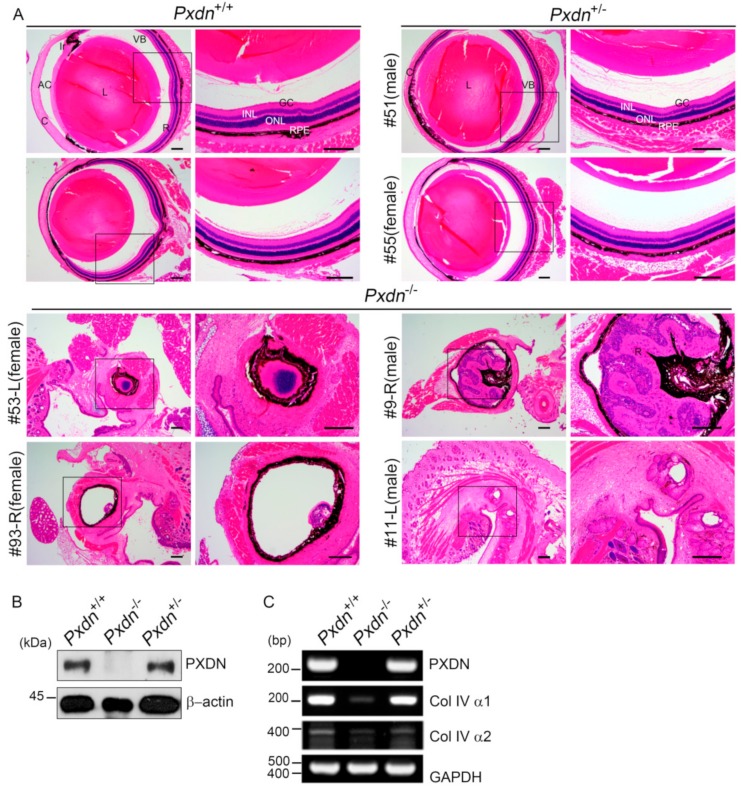
Histological analysis of *Pxdn* KO mice. (**A**) Representative histological data of the eye tissues of the WT, heterozygous, or KO mice (13–15 weeks old). Paraffin-embedded eye sections were stained with hematoxylin and eosin staining (H&E). The eye tissues of heterozygous mice (*n* = 10, male = 5, female = 5) displayed normal anterior segments similar to that of the WT mice (*n* = 6, male = 3, female = 3), whereas the eye tissue of the KO mice (*n* = 6, male = 4, female = 2) exhibited extremely disorganized eye structures or no eyeball structure. Scale bar: 200 μm. (**B**) Western blot analysis of PXDN expression was done using the eye tissue of the mice. β-actin was used as a loading control. (**C**) RT-PCR analyses of PXDN and Col IV α1 and α2 expression in the eye tissues of the WT, KO, and heterozygous mice. A total of 28 cycles for PXDN and Col IV α1 and 33 cycles for Col IV α2 were carried out for the RT-PCR analysis. GAPDH was used as a control.

To analyze histological abnormalities, we carried out hematoxylin and eosin staining (H&E) staining using paraffin-embedded eye sections (Figure 3A). Histological analysis of the eye tissues of the KO mice revealed extremely severe abnormalities. H&E staining displayed that lenses were completely missing or remained in trace. Disorganization of retinal structure, such as retinal folds or rosette-like structures, and retinal dysplasia were also observed. Furthermore, even eyeball formation was absent in the KO mouse (#11-L). To confirm PXDN expression in the eye tissue of the mutant mice, we performed western blot analysis. As shown Figure 3B, the KO mice did not express PXDN, in contrast to the WT and heterozygous mice. When Col IV isoforms (α1 and α2), substrates of PXDN, were examined for gene expression by RT-PCR analysis, the expression levels of both isoforms were reduced only in the eye tissues of the KO mice with ocular developmental defects including missing lenses. Therefore, we concluded that PXDN is important in organization of eyeball structure and lens development during eye development. 

### 2.4. The Pxdn Gene Is Haplosufficient for Visual Function

**Figure 4:**
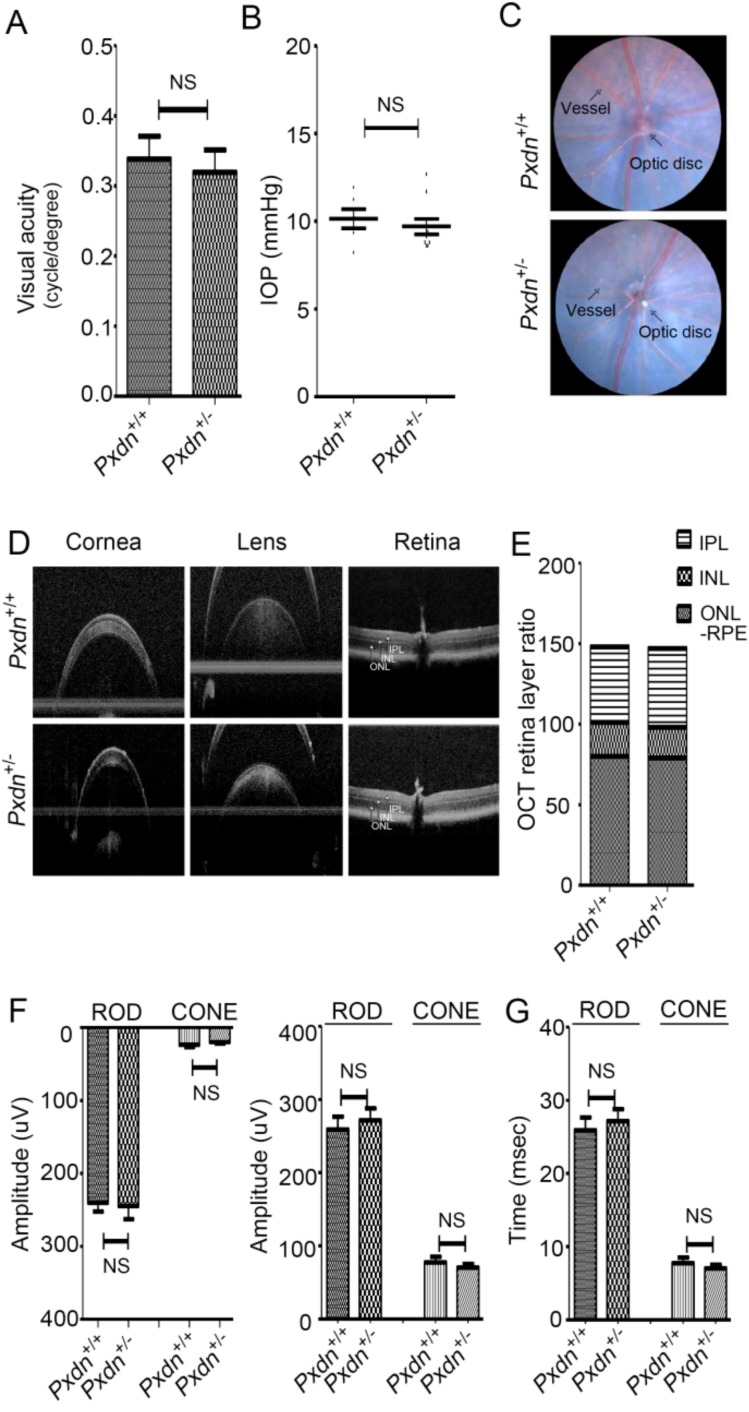
Visual test of the WT and heterozygous mice. (**A**) Visual acuity was measured by optokinetic nystagmus. (**B**) Mouse intraocular pressure (IOP) was measured using tonometry equipment. NS, not significant. (**C**) Fundus photography was used to compare any changes in retina, optic nerve, or retinal vessels pattern between the WT and heterozygous mice. Both groups showed a normal retinal phenotype with the radial pattern of arterioles and venules. (**D**) Representative optical coherence tomography (OCT) cross-section images including cornea, lens, and retina by horizontal meridian of the WT and heterozygous mice. (**E**) The retinal layer and boundary identifications: inner plexiform layer (IPL), inner nuclear layer (INL), and outer nuclear layer (ONL). (**F**) Evaluation of visual function in the mice following electroretinography (ERG) recording. ERG recordings of photopic ERGs and scotopic ERGs from the WT and heterozygous mice were performed. Dark-adapted and light-adapted ERGs were elicited by eight or six different stimulus intensities in rod and cone cells, respectively. ERG a-waves are shown in amplitude of dark-adapted and light-adapted conditions (left). ERG b-waves are shown in amplitude of dark-adapted and light-adapted conditions (right). (**G**) The response time of light sensitivity of retina (*n* = 6/WT and *n* = 10/heterozygote).

There were no differences observed in external morphology, PXDN expression, or Col IV crosslinking level between the WT and heterozygous mice, although deletion of double alleles of the *Pxdn* gene led to no eyeball structures or extremely disorganized eyes. Thus, we were interested in whether a single-allele *Pxdn* gene is sufficient for eye function. To evaluate visual function, we measured visual acuities and intraocular pressure (IOP) for the WT and heterozygous mice (13–15 weeks old) using optokinetic nystagmus and tonometry. There was no difference in visual acuity (Figure 4A) or IOP (Figure 4B) between the WT and heterozygous mice. We also used fundus biomicroscopy to analyze the phenotype of retinas, including the retina, optic nerve, and distribution of retinal blood vessels. We found that both the WT and the heterozygous mice showed a normal retinal phenotype (Figure 4C). By means of optical coherence tomography (OCT), we confirmed normal structure of the cornea, lens, and retina in both the WT and heterozygous mice (Figure 4D). There was no difference in inner plexiform layer (IPL), inner nuclear layer (INL), outer nuclear layer (ONL), or retinal pigment epithelium (RPE) retinal ratio between the two groups according to OCT retinal-layer depth analysis of the WT and heterozygous mice (Figure 4E). The electrical signal stage by stage-light stimulation of rod and cone cells and their response time by means of electroretinography (ERG) in scotopic/photopic condition also did not show any significant differences between the WT and heterozygous mice (Figure 4F,G). Such results indicate that the heterozygous mice had no defect in behavior, morphology, or function in visual acuity compared to the WT mice. Therefore, we concluded that the *Pxdn* gene is haplosufficient for visual function. 

## 3. Discussion 

Because PXDN is known to catalyze the formation of the sulfilimine crosslink that stabilizes the Col IV network for mammalian tissue genesis [19], we suspected there would be embryonic lethality or severe impairment of development in *Pxdn*-null mice. However, unlike our speculation, the *Pxdn* KO mice did not show any apparent external morphological changes compared to those of the wild-type mice except for the abnormal ocular phenotypes, anophthalmia, and severely malformed microphthalmia. Previously, it had been reported that the ENU-induced *Pxdn* mutation that has a premature stop codon (Cys1272X) leads to severe anterior segment dysgenesis and microphthalmia [16]. In humans, the patients with several inherited mutations of the *PXDN* gene also display eye disorders at various levels [14,15]. Such a phenotype shown by PXDN mutations in humans and mice was displayed in the *Pxdn* KO mice. In the *Pxdn* KO mice, deletion of exon1 and its 5′ upstream sequences of the *Pxdn* gene resulted in complete gene inactivation by showing no mRNA/protein expression of PXDN in the tissues. On the other hand, the *Pxdn* mutation with the premature stop codon (Cys1272X) was predicted to cause loss of function of peroxidase enzyme activity with the loss of the von-Willebrand factor type C (vWFC) domain [16]. In their data, an immunofluorescence study showed that PXDN is expressed in the lens and the inner neuroblast layer of mutant eyes at E17.5 with an expression pattern similar to that of wild types. The peroxidase inhibitor phloroglucinol inhibits formation of sulfilimine crosslinks by PXDN at the cellular level [10], and mutagenesis studies have also revealed that peroxidase activity is required for sulfilimine-bond formation by PXDN [20,21]. On the other hand, it cannot be excluded that ECM motifs can have their own function. Thus, it should be clarified in the future whether this point-mutated mouse expresses the truncated form of PXDN with loss of peroxidase activity and vWFC domain, and whether N-terminal ECM motifs can complement PXDN deficiency at some levels in mice. In this study, although *Pxdn*-null mice showed severely defective eye phenotypes, the heterozygous mice with only a single intact allele did not show any defect in structure and visual function of eyes. Thus, we clearly showed that one allele of the *Pxdn* gene is sufficient for eye structure formation and visual function.

Because a number of ophthalmological differences and sequence variants between C57BL/6J and C57BL/6N strains were identified from the comparative phenotypic and genomic sequence analysis [22], it cannot be ruled out that the different genetic backgrounds for the C1272X mutants (C57BL/6J) and the KO mice (C57BL/6N) may contribute to the differences in phenotype. However, the comparison of the general visual functions of the two strains shows only reduced vision in C57BL/6N mice compared with C57BL/6J mice, not reflected relative to differences in lens opacities in both strains. Thus, severe ocular malformation by biallelic deletion of *Pxdn* is not likely strain-dependent, but further studies are needed in future for clarification on this point.

Like the mutant mice with this premature stop codon (Cys1272X), the *Pxdn* KO mice had a white spot at the ventral and/or dorsal region, and their tail color was white in two-thirds of the tip [16]. The previous report noted that this phenotype is reminiscent of some mutations affecting migration of neural crest cells, which also affects eye development [16,23,24]. Thus, it is interesting to figure out how *Pxdn* gene function is correlated with neural crest cell migration.

The lens capsule is a modified BM, which is important for structure, biomechanics, and maintenance of the lens cell phenotype [25]. Some core structural molecules, including Col IV, laminin, and nidogen, provide protective and signaling roles for the mature lens. Col IV point mutants act as dominant negative molecules and affect synthesis and secretion of Col IV or the function of the Col IV network, which leads to defects of the kidneys, esophagus, aorta, and eyes [4]. Therefore, BM defects can lead to damage to cell and tissues, developmental defects, and diseases [19]. The decrease of Col IV expression level in the eye tissue of the *Pxdn* KO mice compared with that of the WT mice may be because of ocular developmental defects including missing lenses, as Col IV is highly expressed in lens capsules [26]. From this study, PXDN deficiency is presumed to be associated with developmental disability in eyes, as they lack the stability of the Col IV network because of the deficiency of sulfilimine crosslinks. The defect in eye phenotype of Col IV mutant mice, such as iris/corneal adhesion, is similar to that of the *Pxdn* mutant mice [16], and the *Pxdn* KO mice showed highly disorganized eye structure or no eyeballs. Therefore, the sulfilimine crosslinks in Col IV may be very important in the development of eye tissue, which requires a complex, precise structure for precise functioning of the eyes. In addition, this fact suggests the importance of the BM integrity generated by PXDN, which is responsible for Col IV network stability. However, it is notable that *Pxdn* gene inactivation by deletion of exon1 and its 5′ upstream sequences leads to no apparent developmental defect in kidney, esophagus, or aorta, which is different from the defects of Col IV mutant mice [4]. A recent study using *Pxdn* KO mice generated by exon 9 deletion also showed no alteration of BM structure in the renal glomeruli or tubules, but reported a finding that reduced Col IV sulfilimine crosslinks reduced renal tubular BM stiffness [27]. This finding corresponds with our finding suggesting the importance of BM integrity driven by PXDN. 

Considering that the sulfilimine bond is presumed to provide the mechanical strength for the Col IV network, it is still questionable as to why it is important in the development of the eyes. Anophthalmia, the most severe phenotype, is associated with mutations of the *Rax, Sox2, Lhx2, BMP-4,* and *BMP7* genes [28]. Complete inactivation of these genes also displays embryonic lethality because of broad impacts on development. *Pax-6* homozygous mutants, the classical model for anophthalmia in the mouse, do not develop eyes, but die perinatally because of other developmental defects, including those of the central nervous system [29,30]. In the future, it will be interesting to address why the contribution of PXDN in tissue genesis of mice is markedly significant only for the eyes, but not for other organs, such as the kidneys and vessels. 

From this study, we can suggest that the *Pxdn* gene is haploid sufficient for normal eye development and visual function, providing evidence that PXDN is essential for eyeball development. It will be also interesting to investigate in the future whether PXDN is correlated with the pathogenesis of glaucoma and cataract in adult eyes after normal eye development. 

## 4. Materials and Methods 

### 4.1. CRISPR/Cas9 for Pxdn Gene Knockout

We purchased SpCas9 protein and sgRNAs to generate *Pxdn* knockout mice from Macrogen, Inc. (Seoul, Korea). To knock out the *Pxdn* gene (NC_000078.6), we designed two sgRNAs (1 (5′ UTR gR3), GTCGCTAGGTTCAGACACCAAGG; 2 (intron1-2-gR2), CTCAACTTTCTCTCCACTCGGGG) to target the exon1 and its 5′ upstream sequences of the *Pxdn* gene and validated them using the T7E1 in vitro cleavage reaction of template DNAs, which were amplified by PCR (F1:5′-TGTGGGAACAGTGGTGGGCA-3′ and R1: 5′-GTGAACAGCAGCGGAGGAG-3′, F2: 5′-CAGGGGTACTGGTTTGGAGA-3′ and R2: 5′-CCAAGCAAAGAAAAAGCTCCT-3′). Briefly, the amplified template DNA was incubated for 90 min at 37 °C with Cas9 protein (20 nM) and sgRNA (40 nM) in 1× NEB 3 buffer. Reactions were stopped with 6× stop solution containing 30% glycerol, 1.2% SDS, and 100 mM EDTA. Cleavage activity was confirmed by electrophoresis of the reaction mixture.

### 4.2. Generation of Pxdn Gene Knockout Mice 

*Pxdn* knockout mice were generated by Macrogen, Inc., and were interbred and maintained in a pathogen-free condition at Macrogen, Inc. (Seoul, Korea). All animal experiments were performed in accordance with the Korean Food and Drug Administration (KFDA) guidelines. Protocols were reviewed and approved by the Institutional Animal Care and Use Committees (IACUC) of Macrogen, Inc. (MS-2016-01(5 October 2016)). All manipulations were conducted with the Institutional Animal Care and Use Committee approval. Briefly, pregnant mare serum gonadotropin (PMSG) and human chorionic gonadotropin (hCG) were injected into C57BL/6N female mice. After 48 h, these female mice were mated with C57BL/6N stud male mice. Next day, virginal plug-checked female mice were sacrificed and fertilized embryos were harvested. The mixture of sgRNA and SpCas9 protein was microinjected into one-cell embryos, and microinjected embryos were incubated at 37 °C for 1–2 h. Then, 14 to 16 injected one-cell-stage embryos were transplanted into oviducts of pseudopregnant recipient mice (ICR). After F0 mice were born, genotyping was done by direct PCR and sequencing methods using tail-cut samples (SF3 primer, 5′-TGGCAGAACTGGGTAACTCC-3′; and SR3 primer, 5′-CAATCTCCCCCAGAGGAAGT-3′). Among of the founders with altered sequences, we selected F0 mice with deletion of exon1 and its 5′ upstream sequences of *Pxdn*.

### 4.3. Genotyping 

Genomic DNA was isolated from the lung tissues of each mouse using an MGmed tissue kit (MGmed, Seoul, Korea). Genotyping was done by PCR amplification using the following primers: SF1, 5′-AAGCAGAATCAGGGATCCAA-3′; and SR2, 5′-AGCCGATGGGAGTACACTTG-3′. The PCR products were visualized by agarose gel electrophoresis; a 1096 bp fragment was amplified in the wild-type mice, both 1096 bp and 336 bp fragments in the heterozygous mice, and a 336 bp fragment in *Pxdn*-null mice. 

### 4.4. RT-PCR Analysis

Expression of PXDN in the tissues was confirmed by RT-PCR analysis. Total RNA was extracted from the tissues by Trizol reagent (Invitrogen, Carlsbad, CA, USA). We performed cDNA synthesis using the SuperScript synthesis system for RT-PCR (Enzynomics, Daejeon, Korea) according to the manufacturer’s instructions. The primer sets used for PXDN were as follows: for 5′ UTR-exon 19, forward, 5′-GACCGGAGGGCTCAGTTG-3′ and reverse 5′-ACACGGGTGATGTTGTCTGA-3′; for exon 10–14 (IgC2 3-4 domain), forward 5′-CGCGCGGCAGCCATATGGCCCTTCTCAGTTCACT-3′ and reverse 5′-GTTAGCAGCCGGATCCTCAATTCACACTGAGTACCATGCT-3′; for exon 17–19 (peroxidase domain), forward 5′-GGCTGTATGGCTCGACTCTC-3′and reverse 5′-ACACGGGTGATGTTGTCTGA-3′; for 3′ UTR, forward 5′-GGCTAGGGAGGAAGACCTCA-3′ and reverse 5′-CCTGGGCAATGAGGCTGTAA-3′; for GAPDH, forward 5′-CATGACCACAGTCCATGCCATCACT-3′ and reverse 5′-TGAGGTCCACCACCCTGTTGCTGTA-3′. The following primers were used to measure the mRNA expression level of Col IV: forward 5′-GCCAAGTGTGCATGAGAAGA-3′ and reverse 5′-AGCGGGGTGTGTTAGTTACG-3′ for Col IV α1, forward 5′-CCGATTCCACGAGCCCCTT-3′ and reverse 5′-CTCCTTTCTCCGGGTAGCAC-3′ for Col IV α2. PCR amplification for PXDN mRNA expression was done under the following conditions: 95 °C for 1 min, followed by 28–33 cycles of 1 min at 95 °C, 1 min at 56–64 °C, and 1–4 min at 72 °C. The PCR product was loaded in 1–2% agarose gel mixed with SYBR safe dye (Invitrogen). 

### 4.5. Preparation of Anti-Mouse PXDN Antibody

The recombinant peroxidasin IgC2 (3–4) subdomain (amino acids 428–609) of mouse PXDN was prepared for antibody production. A 546 bp DNA fragment encoding amino acids spanning from Ala (428) to Asn (609) of mouse PXDN was amplified by PCR using mouse lung cDNA, *Pfu* polymerase, and primers (forward: 5′-CGCGCGGCAGCCATATGGCCCTTCTCAGTTCACT-3′, reverse: 5′-GTTAGCAGCCGGATCCTCAATTCACACTGAGTACCATGCT-3′) and subcloned between the *Nde*I and *Bam*HI sites of pET-15b (Novagen, Madison, WI, USA). Then, the recombinant protein was expressed in *E. coli* BL21 (DE3) and purified from the cell pellet as described previously [31]. Polyclonal antibody was raised in rabbits against the purified recombinant protein (Abfrontier, Seoul, Korea).

### 4.6. Western Blot Analysis

For protein preparation, the tissues were dissected and lysed using lysis buffer (50 mM Tris (pH 8.0), 150 mM NaCl, 1% Triton X-100, 1% sodium deoxycholate, 0.1% SDS, 1 mM Na_3_VO_4_, 50 mM NaF, 1 mM EDTA, 1 mM EGTA, 2 mM PMSF, 1 μg/mL pepstatin, and protease inhibitor mix (Roche, Basel, Switzerland)). The lysate was centrifuged at 14,000 rpm for 30 min, and then the supernatant was subjected to 8% SDS-PAGE. Separated proteins were transferred to a nitrocellulose membrane, and the membrane was blocked with 5% skim milk. Blots were incubated with polyclonal anti-mouse PXDN antibodies raised against recombinant IgC2 (3–4) subdomain, and then with horseradish peroxidase (HRP)-conjugated secondary antibodies. GAPDH or β-actin was used for a loading control. We visualized the immunoreactive bands using a chemiluminescent substrate (ECL kit) (Amersham, Amersham, United Kingdom).

### 4.7. Detection of NC1 Sulfilimine Crosslink

We assessed the level of NC1 domain sulfilimine crosslinking in Col IV according to a procedure reported previously [32]. Briefly, the lung tissues were lysed in hypotonic lysis buffer (10 mM CaCl_2_, 50 mM HEPES, pH 7.4, 0.1 mM benzamininde hydrochloride, 25 mM 6-aminocaproic acid, and 1 mM PMSF). The tissue lysate was digested with 0.5 mg/mL collagenase (Gibco) at 37 °C for 18 h and then subjected to SDS-PAGE under reducing conditions followed by western blotting. Crosslinked dimeric and un-crosslinked monomeric NC1 domains were detected with anti-Col IV α2 antibody (ChondrexInc, Redmond, WA, USA).

### 4.8. Hematoxylin and Eosin Staining 

For the histological examination, the eye specimen was fixed with 4% Davidson’s solution for 24 h with gentle shaking. Fixed samples were paraffin-embedded and cut into 5 μm sections using a microtome (RM2335, Leica, Wetzlar, Germany). Hematoxylin and eosin staining (H&E) was done after a day of sectioning. For H&E staining, paraffin sections were deparaffinized and then hydrated in a descending grade of ethanol. Next, we stained sections with 0.1% Mayer’s hematoxylin for 10 min and 0.5% eosin in 95% EtOH. After staining with hematoxylin and eosin, the washing steps were immediately and sequentially done as follows: dipped in distilled water until eosin stopped streaking, dipped in 50% EtOH 10 times, dipped in 70% EtOH 10 times, and incubated in 95% EtOH for 30 s and 100% EtOH for 1 min. Then, samples were covered with mount solution (6769007, Thermo Scientific, Waltham, MA, USA) and examined under the light microscope (BX43, OLYMPUS, Shinjuku, Japan).

### 4.9. Optokinetic Nystagmus (OKN)

All mice for visual function tests were in accordance with protocols approved by the Institutional Animal Care and Use Committee (IACUC: 2018-0145) of Yonsei University. All animals were handled according to the principles of the Association for Research in Vision and Ophthalmology statement. We assessed spatial frequency thresholds (i.e., visual acuity) by optokinetic nystagmus (OKN) using a virtual optokinetic system (OptoMotry, Cerebral Mechanics, Medicine Hat, Alberta, Canada). A video camera located on the ceiling of the device recorded the images and sent them to the connected computer. The clockwise movement was to track the movement of the left eye, and the counterclockwise movement tracked the reaction of the right eye of the mouse. The experimenter judged whether the head and body of the mouse were tracking according to the direction in which the drift grid was rotating. If the presence of the tracking was unclear or absent, the retry was resumed, ignoring large repositioning and grooming movements. The maximum spatial frequency capable of driving head tracking was determined.

### 4.10. Tonometry

We anesthetized mice with an intraperitoneal injection of xylazine (10 mg/kg; Rompun, Bayer Animal Health, Leverkusen, Germany) and zolazepam and tiletamine (30 mg/kg; Zoletil 50, Vibrac, Carros, France). Intraocular pressure (IOP) was measured using a rebound tonometer (Icare TONOLAB tonometer, Colonial Medical Supply, Franconia, NH, USA). IOP measurements were taken according to the manufacturer’s instructions. One trial result was given after six consecutive measurements, and the mean of consecutive trials was used for analyses.

### 4.11. Optical Coherence Tomography (OCT) 

We anesthetized mice with an intraperitoneal injection of xylazine (10 mg/kg; Rompun, Bayer Animal Health) and zolazepam and tiletamine (30 mg/kg; Zoletil 50, Vibrac, Carros, France). The pupils were dilated with 0.5% tropicamide and 0.5% phenylephrine mixed eyedrop (Mydrin-P, Santen Pharmaceutical Co, Ltd., Osaka, Japan). Optical coherence tomography (OCT) scans were taken using the Eyemera OCT (IISCIENCE, Busan, Korea). The cornea was lubricated with hypromellose 2.5% (Goniovisc, Hub Pharmaceuticals LCC, Roncho Cucamonga, CA, USA). After placing the mouse in front of the OCT lens and focusing on the retina, fundus photography and OCT scan were performed. Retinal thickness was measured using InSight-Animal OCT Segmentation Software (Phoenix Research Labs, Pleasonton, CA, USA). Each scan for cornea, lens, and retina centered on the optic nerve was obtained.

### 4.12. Electroretinography (ERG)

We performed ERG analysis using Micron Ganzfeld ERG (Phoenix Research Labs, Pleasanton, CA, USA). Mice were dark-adapted at least 12 h before the experiment for scotopic testing (rod-cell response). After anesthesia, the pupil was dilated as previously described. Once the pupil was fully dilated, we applied hypromellose 2.5% (Goniovisc) and inserted the electrodes. ERG was recorded as Ganzfeld ERG according to the standard protocol of the manual instruments. Scotopic ERG was obtained with increasing flash intensity in the range of −1.7 log cd/s/m^2^ to 1.9 log cd/s/m^2^. Mice were light-adapted for 15 min prior to the cone-cell response experiment. Photopic ERG was done with a flash intensity in the range of −0.5 log cd/s/m^2^ to 4.1 log cd/s/m^2^. A total of 10 responses to light stimuli were averaged. The implicit times of a-wave (as a measure of photoreceptor function), b-wave (as a measure of bipolar cell function), amplitude, and rod- and cone-cell response were determined.

### 4.13. Data and Statistical Analysis

Data were presented as means ± SEM of representative experiments. All the in vivo visual experiments were performed at least six times. Student’s *t*-test was used to calculate statistical difference between the two groups.
